# Correcting for bias in the selection and validation of informative diagnostic tests

**DOI:** 10.1002/sim.6413

**Published:** 2015-02-01

**Authors:** David S Robertson, A Toby Prevost, Jack Bowden

**Affiliations:** a MRC Biostatistics Unit, Institute of Public HealthCambridge, UK; b King's CollegeLondon, UK

**Keywords:** diagnostic tests, group sequential design, family history, uniformly minimum variance unbiased estimator

## Abstract

When developing a new diagnostic test for a disease, there are often multiple candidate classifiers to choose from, and it is unclear if any will offer an improvement in performance compared with current technology. A two-stage design can be used to select a promising classifier (if one exists) in stage one for definitive validation in stage two. However, estimating the true properties of the chosen classifier is complicated by the first stage selection rules. In particular, the usual maximum likelihood estimator (MLE) that combines data from both stages will be biased high. Consequently, confidence intervals and ***p***-values flowing from the MLE will also be incorrect. Building on the results of Pepe *et al*. (SIM **28**:762–779), we derive the most efficient conditionally unbiased estimator and exact confidence intervals for a classifier's sensitivity in a two-stage design with arbitrary selection rules; the condition being that the trial proceeds to the validation stage. We apply our estimation strategy to data from a recent family history screening tool validation study by Walter *et al*. (BJGP **63**:393–400) and are able to identify and successfully adjust for bias in the tool's estimated sensitivity to detect those at higher risk of breast cancer. © 2015 The Authors. *Statistics in Medicine* published by John Wiley & Sons Ltd.

## 1. Introduction

The development and validation of an informative diagnostic test for a medical condition is of great use for clinicians. This process is well described in the literature if only a single diagnostic variable is studied. However, there are often multiple candidate classifiers that show potential as diagnostic tools, and it may also be unclear if any will offer an improvement compared to current technology. The challenge is to identify the *most* promising diagnostic test and then to correctly validate its properties.

It is in the context of biomarker research that this challenge is particularly evident, where new technological advancements have led to an abundance of biomarker discovery studies and a huge number of candidate markers, for example, in colorectal cancer 1 and prostate cancer 2. Guidelines have also been established for the discovery and validation of potential biomarkers 3.

The development of questionnaires for diagnosis is a parallel endeavour to biomarker discovery and validation. There will be a set of possible questions, with each considered a candidate classifier. In particular, questions about the family history of a disease are simple and cheap to measure when compared with genetic or biomarker variables. They can also provide the bulk of a diagnostic or risk prediction tool's classification ability, despite the discovery of many genetic markers 4.

To make efficient use of resources, a sequential procedure is a natural choice for the selection and validation of diagnostic tests. This is particularly the case for biomarkers, due to the high false discovery rate – despite showing initial promise, the majority of markers will not subsequently perform well enough compared with an existing test to be considered for further development. Also, many biomarker studies rely on stored biological samples, and there is a need to preserve specimen resources 5. Hence, group sequential designs have been proposed that allow for early stopping because of poor marker performance 5,6. In these settings, the simplest (two-stage) group sequential design has been proposed; whereby the discovery and validation phases are separated by a single interim analysis.

Estimating the performance of the chosen classifier is complicated by the first stage selection rules. A candidate classifier will have to perform well in the first stage in order to proceed to the validation stage, which will lead to overly optimistic estimates. In particular, the usual maximum likelihood estimator (MLE) that combines data from both stages will be biased high. Hence, hypothesis-testing procedures using the MLE will have incorrect *p*-values, with an inflation of the type I error rate. Furthermore, confidence intervals will have coverage probabilities that can be well below the nominal level.

There are obvious parallels in this endeavour with multi-arm adaptive clinical trials of pharmaceutical treatments, where a promising single treatment or treatment dose is selected in a preliminary phase for a subsequent confirmatory analysis against standard therapy. Specific examples include seamless designs 7,8 and drop-the-losers trials 9. In this domain, the issues of bias and type I error inflation are well understood. Many methods exist to adjust for bias 10–13 and to ensure correct hypothesis testing 9,14 because of demands of regulatory authorities when making licensing decisions based on trial evidence.

Bias and type I error are also important in the diagnostic test setting. Like pharmaceutical drugs, they are marketed and sold to the healthcare industry on the basis of their (claimed) clinical utility. They can have a pivotal role in guiding the treatment plan of patients 15. Hence, diagnostic tests are subject to rigorous approval pathways by regulatory authorities.

In the spirit of Cohen and Sackrowitz 13, an efficient unbiased estimator can be obtained by taking the unbiased stage two data and conditioning on a complete, sufficient statistic – a technique known as Rao–Blackwellisation. By the Lehmann–Scheffé theorem, this will give the uniformly minimum variance conditionally unbiased estimator (UMVCUE). In a similar vein, uniformly most powerful conditionally unbiased (UMPCU) hypothesis tests have also been developed 14,16. The ‘condition’, in each case, is that the single treatment has been selected from many at stage 1 and carried forward to the validation stage.

The rationale for this continued conditional perspective is that estimation is only important if a promising classifier is actually identified. Indeed, when a study appropriately terminates early, the candidate classifiers are then deemed inadequate and further estimation of their performance is not needed. This viewpoint is demonstrated in a number of recent examples 5,6,17.

An alternative argument for the use of conditional estimators and confidence intervals is that we are essentially combining a discovery and validation study into a single, two-stage design. In this setting, the conditional estimators offer properties that are analogous to what would be observed if an independent validation study was completed, but are more efficient because they utilise the data from the discovery phase.

In this research article, we focus on finding the UMVCUE for the chosen classifier's sensitivity (or true positive rate) when the candidate classifiers are dichotomous. For example, this could correspond to the absence/presence of a biomarker or a ‘yes’/‘no’ question in a questionnaire. Once the UMVCUE is found, we then construct confidence intervals for the estimated sensitivity.

Pepe *et al*., 5 considered a two-stage study for a *single* dichotomous diagnostic biomarker, with early stopping for futility. They derived the UMVCUE and described bootstrapping schemes to estimate confidence intervals for the sensitivity. Prior to this, Tappin 18 provided methodology to find the UMVCUE when selecting from multiple dichotomous classifiers (provided that ties were broken according to a pre-specified ordering) but without the option of stopping for futility or the construction of confidence intervals. This latter issue was addressed by Sill and Sampson 16, who showed how to construct *exact* confidence intervals when there are multiple candidate classifiers to choose from in the first stage.

We extend the above approaches for finding the UMVCUE and exact confidence intervals by allowing the following: (i) generalised rules for ranking the candidate classifiers; (ii) arbitrary (fixed) futility thresholds for each classifier; and (iii) unequal stage one sample sizes.

In Section 3, we describe the model framework and show how to derive the UMVCUE and construct exact confidence intervals. We then carry out a simulation study in Section 4 to investigate their properties. In Section 5, we apply our inferential technique to a recent family history screening tool validation study by Walter *et al*. 19 and conclude with a discussion in Section 6. However, we first describe the data that served as motivation for this work.

## 2. Motivation: The family history questionnaire study

Walter *et al*. 19 implemented a two-stage diagnostic validation study in 10 general practices across eastern England. The aim was to develop a brief self-completed family history questionnaire (FHQ) that accurately identified people at higher risk of diabetes, ischaemic heart disease (IHD), breast cancer and colorectal cancer. This self-completed FHQ would be a cheaper and simpler alternative to the current gold standard in-depth interview.

There were 1147 participants recruited into the study, with 618 in stage 1 and 529 in stage 2. This sample size was chosen to give at least 90% power to detect whether those answering ‘yes’ to a question would have a different risk from those answering ‘no’. Overall, 32% were at an increased risk of one or more of the conditions, as assessed by the three-generational gold standard pedigree collected by trained research nurses.

In stage 1 of the analysis, the FHQ consisted of 12 questions (14 including sub-questions). Questions that were sufficiently predictive of increased risk for each condition were identified by the following procedure:

Test for significance of questions using (a two-sided) Fisher's exact test with *p* < 0.05.
Retain the significant question with the greatest balanced accuracy (defined as the arithmetic average of the sensitivity and specificity).
Exclude each significant question if, in combination with the most accurate question, there was no significant improvement in prediction as assessed by a likelihood ratio test with *p* < 0.10.
If necessary, assess further combinations of the remaining significant question using multiple logistic regression.

Questions 4a, 4b, 9a and 9b were not considered in the above analysis by Walter *et al*. because of a small number of positive responses.

Six questions (questions 2, 3, 6, 8, 10 and 11) were taken into the brief FHQ, which was tested on the additional 529 subjects in stage 2. No significant differences in sex, age or prevalence of increased risk for the conditions were found between the participants in stages 1 and 2.

Finally, to validate the retained questions, a *χ*^2^-test was used to compare the sensitivity and specificity between the two stages for each condition. Because there were non-significant differences (*p* > 0.05) for all conditions, the data from both stages were then pooled to give an overall assessment of the brief FHQ. In particular, combined results were given for the sensitivity and specificity of the selected questions.

**Figure 1:**
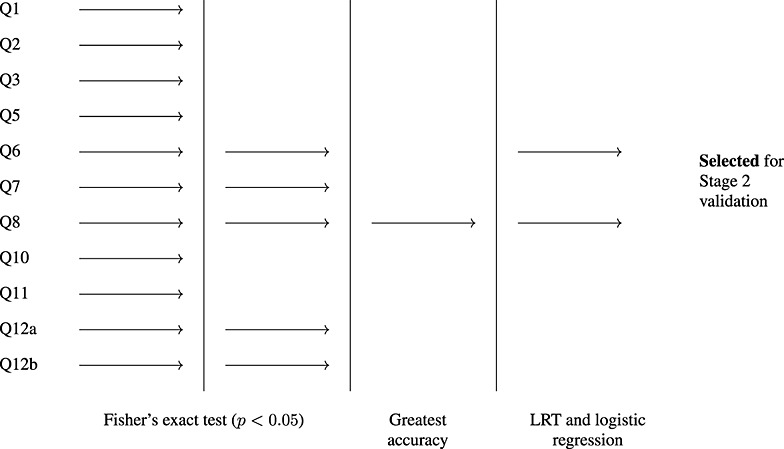
Schematic of the stage 1 selection process for identifying sufficiently predictive questions for breast cancer.

A schematic of the stage 1 selection process for breast cancer is given in Figure 1. Question 8 was the significant question with the highest balanced accuracy and was selected for further validation in stage 2. Question 6 was also selected on the basis of a likelihood ratio test.

Through its use of a two-stage design and a complex interim selection rule, the development of the brief FHQ has clear parallels to a biomarker discovery and validation study. Therefore, it inherits many of the same issues of bias and type I error inflation. In the next section, we describe how to derive efficient unbiased point estimates and confidence intervals under general selection rules, for which the FHQ study is a special case.

## 3. General framework for the uniformly minimum variance conditionally unbiased estimator

### 3.1 Model description

Suppose there are *K* candidate binary classifiers, each taking values in {0,1}. For example, this could correspond to a set of *K* candidate diagnostic biomarkers or a questionnaire with *K* ‘yes’/‘no’ questions. The aim is to select the classifier that performs ‘best’ (as defined below), subject to passing a ‘fixed’ threshold and then to estimate its sensitivity. To do so, we perform a two-stage validation study.

In the first stage, each classifier *i* is tested on a population that contains *n*_1*i*_ known case subjects. These could be disease cases or those that have been classified as a case by some gold standard test. Ideally, the classifiers could be all tested on the same population; hence, the *n*_1*i*_ would all be equal. However, commonly, the number of case subjects will vary between the classifiers. This could be because of missing data or because the classifier is not applicable to all subjects (e.g. gender-specific questions).

Let *X*_*i*_ denote the number of true positives for classifier *i*. Hence, we assume that we have *K* independent binomial variables *X*_*i*_∼Bin(*n*_1*i*_,*s*_*i*_) for *i* = 1,…,*K*, where *s*_*i*_ is the true *sensitivity* for the *i*^th^ classifier and where sensitivity is defined as Prob(positive test ∣ subject diseased).

Each classifier has an associated fixed threshold that the number of true positives must pass in order to be considered further in stage 1. That is, for each *i*∈{1,…,*K*} there is a fixed cut-off *c*_*i*_, and we require 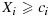 or else classifier *i* is dropped for ‘futility’. For example, if there already exists a classifier with known sensitivity 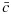, then we might set 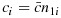. If all the classifiers fail to pass their respective fixed thresholds, then the whole study is terminated early.

Suppose that *L* > 0 classifiers pass their fixed threshold. Let 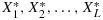 denote the number of true positives, where the relabelling preserves the original ordering of the labels (this is important for breaking ties). The *L* classifiers are then ranked from ‘best’ to ‘worst’ using a pre-specified function 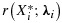, where the ***λ***_*i*_ are constants associated with classifier *i*.

Thus, classifier *i* is ranked above classifier *j* if 
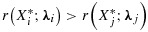
. If there is a tie, 
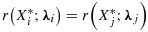
, we choose the classifier with the smallest index. This allows us to rank the classifiers in *a priori* order of importance. For instance, we might pre-rank the classifiers on the basis of evidence from previous studies, biological plausibility or simply the cost of measurement. A fully Bayesian approach is also possible, where classifiers are ranked using the posterior distribution of the *s*_*i*_, given the specification of suitable priors. Note that the method used for breaking ties is important. For example, Tappin 18 showed that if ties are broken by randomisation, then, in fact, no UMVCUE exists.

We also require 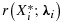 to induce the following inequalities on the 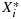:

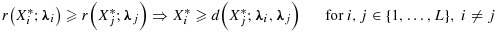
 where 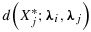 is a function that only depends on 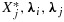 and not on 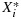. Hence, there is equality if and only if there is a tie in the rankings. Note that 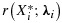 need not to be explicitly defined by the study organisers, as complex selection rules can be reverse engineered to conform to this set up, as we show for the FHQ study.

As an example of the above formulation, consider ranking the classifiers by their estimated sensitivities and, hence, ***λ***_*i*_=*n*_1*i*_ and 
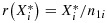
. This induces the following inequality:

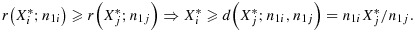
 At the end of stage 1, the classifier with the highest ranking (that has passed its fixed threshold) is then selected for further validation in stage 2. Let *M* be the index of this chosen classifier. In the second stage, the selected classifier from stage 1 is tested on a population containing *n*_2_ additional cases, where *n*_2_ is a constant that does not depend on 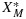. Let *Y* denote the number of true positives in these *n*_2_ additional observations. Note that *Y* ∼ Bin(*n*_2_,*s*_*M*_), independently of 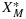.

After the end of the study, we estimate the sensitivity *s*_*M*_ of the selected classifier. The naïve estimator (MLE) for *s*_*M*_ using data from both stages is

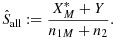
 This estimator is biased high, because it does not take into account the first stage selection procedures and so 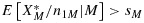.

An unbiased estimator 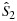 can easily be found by just using the stage 2 data, where 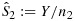. However, given the smaller sample size, then this estimator suffers from lower precision. Hence, we look for an unbiased estimator that utilises data from both stages.

### 3.2 Deriving the uniformly minimum variance conditionally unbiased estimator

In this section, we extend the arguments of Pepe *et al*. 5 and Tappin 18 to find the UMVCUE for the parameter of interest *s*_*M*_.

Let (*i*_1_,*i*_2_,…,*i*_*L*_) denote the vector of indices of the *L* classifiers 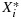 after they have been ranked, with ties being decided by choosing the smaller index. Hence, *M* = *i*_1_ is the index of the selected classifier.

In what follows, we drop the * superscript for notational convenience. We drop the constants ***λ*** from the arguments of the functions *r* and *d* as well.

In Appendix A.1, we show that a complete and sufficient statistic for (*s*_1_,*s*_2_,…,*s*_*L*_) is ***Z*** = (*Z*_1_,*Z*_2_,…,*Z*_2*L*_), where

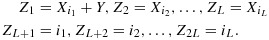
 Let *ψ*(*i*) denote the ranking of the *i*^th^ classifier, and *Q* the event

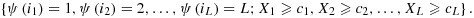
 Then by the Lehmann–Scheffé theorem, 
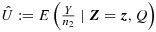
 is the UMVCUE for *s*_*M*_ under *Q*.

Now, following the idea of Pepe *et al*. 5, note that conditional on 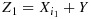, the distribution of *Y* is hypergeometric: *Y*∣*Z*_1_∼Hyper(*z*_1_,*n*_1*M*_+*n*_2_−*z*_1_,*n*_2_), which can be re-expressed (for notational convenience) as *Y*∣*Z*_1_∼Hyper(*n*_2_,*n*_1*M*_,*z*_1_). That is,

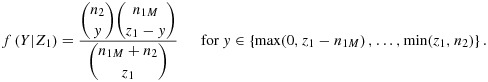
 The conditional density *f*(*Y*∣***Z***,*Q*) is essentially the same, except that the support of *Y* is further restricted by *Q*. There is the ranking condition inequality 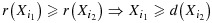 and the fixed threshold condition 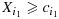.

The precise way that *Y* is additionally restricted under (***Z***,*Q*) is given below.


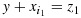


If *i*_1_>*i*_2_⇒ no tie in the ranking is possible 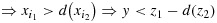


If *i*_1_<*i*_2_⇒ a tie is possible 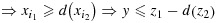



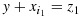
 and 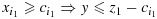


The formula for the UMVCUE (assuming *L* > 1) is then as follows.

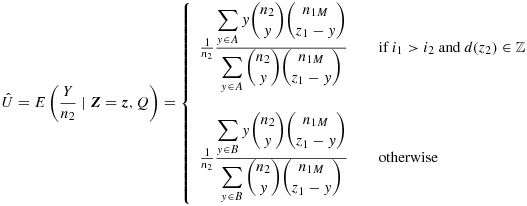
7 where

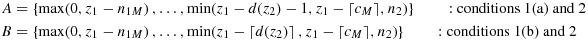
 and ⌈*x*⌉ is the ceiling function acting on *x*.

Note that if the summation over *y* goes up to *n*_2_ (so either max(*A*) = *n*_2_ or max(*B*) = *n*_2_), then, in fact, 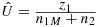, which is just the usual MLE 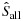. This makes it clear when the stage 1 selection exerts no biasing effect at all.

If *L* = 1, then the dependence on *Z*_2_ disappears, and we are left with the simpler formula below.

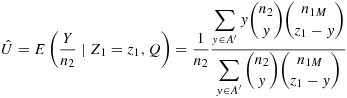
 where *A*^′^={max(0,*z*_1_−*n*_1*M*_),…, min(*z*_1_−⌈*c*_*M*_⌉,*n*_2_)}.

### 3.3 Constructing confidence intervals

After calculating a point estimate for *s*_*M*_ at the end of the study, it is natural to seek a confidence interval as well. In this section, we describe two schemes for generating confidence intervals.

#### 3.3.1 Nonparametric bootstrap

Firstly, we adapt the nonparametric bootstrap procedure originally used by Pepe *et al*. 5. Given trial data ***Z***, the procedure follows the resampling schema below.

Resample the first stage data for the selected classifier *M* = *i*_1_ (with replacement). This gives a bootstrapped number of true positives 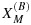.

If 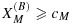 and 
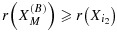


Resample the second stage data (with replacement), giving a bootstrapped number of true positives *Y*^(*B*)^.

Calculate the UMVCUE 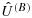
from equation , using 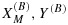
and the original observed value 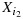.

These steps are then repeated for a large value of *B*, so that there are enough sampled values of 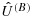 to accurately assess its sampling distribution. The *α*/2 and (1 − *α*/2) empirical quantiles are then used as the (1 − *α*)*%* confidence interval. Bootstrapped confidence intervals for the naïve estimators 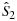 and 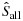 are also immediately available following this procedure.

#### 3.3.2 Sill–Sampson approach

Alternatively, we can adapt the approach used by Sill and Sampson 16, who found *exact* likelihood-based confidence intervals for *s*_*M*_ in the context of two-stage adaptive clinical trial. The derivation is similar to that in the work of Sill and Sampson  16, but we remove the control arm and also additionally allow for early stopping for futility and unequal first stage sample sizes. See Appendix A.2 for further details.

Defining 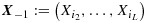, then the conditional distribution used to find the confidence intervals is

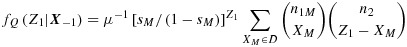
 where

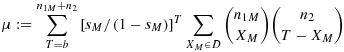
 is the normalising constant and

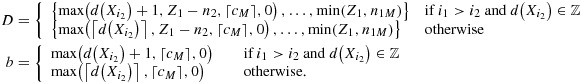
 Suppose we observe *Z*_1_=*Z*_obs_. To construct the (1 − *α*)*%* confidence interval for *s*_*M*_, use the following functions:

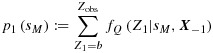
 and

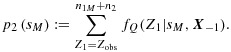
 Bounds for a two-sided (1 − *α*)*%* confidence interval [*Δ*_1_,*Δ*_2_] can then be found by solving the equations *p*_2_(*Δ*_1_) = *α*_1_ and *p*_1_(*Δ*_2_) = *α*_2_ respectively, where *α*_1_+*α*_2_=*α*.

The original Sill–Sampson approach sets *α*_1_=*α*_2_=*α*/2, but this does not (in general) give the shortest confidence interval. We also experimented with choosing *α*_1_ and *α*_2_ to minimise the confidence interval length, which we refer to as ‘optimised’ Sill–Sampson confidence intervals.

#### 3.3.3 Clopper–Pearson approach

In order to see how the Sill–Sampson approach compares with using confidence intervals for the MLE, we use the well-known Clopper-Pearson method 20. This uses the likelihood of the usual MLE to construct exact confidence intervals. Hence, the Sill–Sampson and Clopper–Pearson approaches are both likelihood based, but only the first takes into account the selection rules.

The Clopper–Pearson approach is as follows. Suppose we observe *Z*_1_=*Z*_obs_. Then to construct the (1 − *α*)*%* confidence interval for *s*_*M*_, use the following functions:

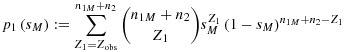
 and

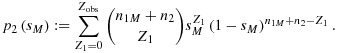
 Bounds for a two-sided (1 − *α*)*%* confidence interval [*Δ*_1_,*Δ*_2_] can then be found by solving the equations *p*_2_(*Δ*_1_) = *α*/2 and *p*_1_(*Δ*_2_) = *α*/2 respectively.

## 4. Simulation studies

We now perform a simulation study using a typical trial design. Consider a two-stage trial conducted on *K* potential diagnostic biomarkers, where the interest is in finding the biomarker with the highest sensitivity. In stage 1, the *i*^th^ biomarker is tested on a population that contains *n*_1*i*_ known case subjects, where the *n*_1*i*_ are not necessarily identical.

Suppose there already exists a biomarker with known sensitivity 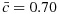. Hence, the fixed cut-off for biomarker *i* is set to *c*_*i*_=0.70*n*_1*i*_. The biomarkers that satisfy 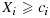 are then ranked by sensitivity, giving *r*(*X*_*i*_) = *X*_*i*_/*n*_1*i*_ and *d*(*X*_*j*_) = *n*_1*i*_*X*_*j*_/*n*_1*j*_. Finally, the selected biomarker (with label *M* = *i*_1_) is taken forward to stage 2, where it is tested on an additional population with *n*_2_=50 case subjects.

### 4.1 Point estimation

**Figure 2:**
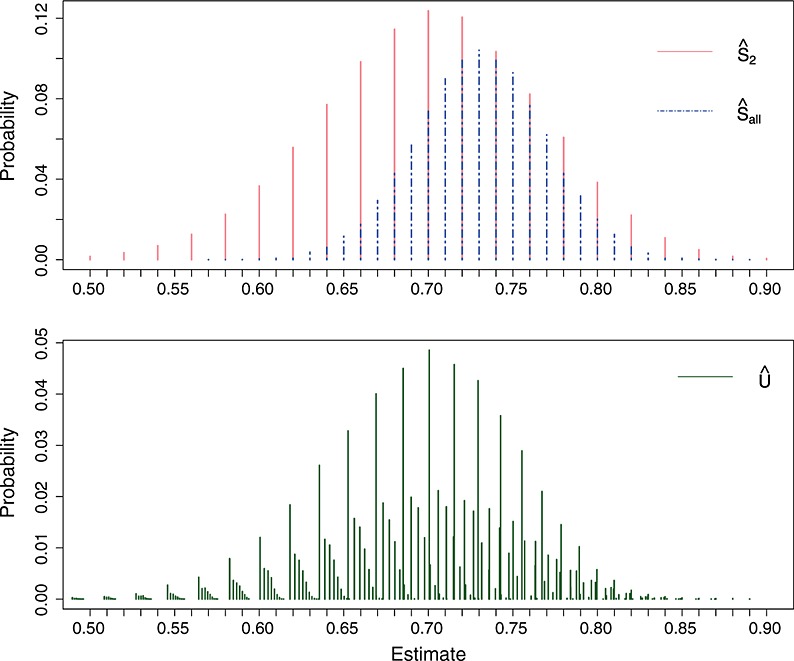
Probability mass functions of the estimators for *S* = (0.70,0.70,0.70), *n*_1_ = (50,50,50), *c* = 0.70*n*_1_ = (35,35,35) and *n*_2_=50. Each mass function is based on 100,000 simulations.

To start with, consider a simple simulation with *K* = 3 biomarkers with equal true sensitivities ***S*** = (0.70,0.70,0.70). Each biomarker is tested on the same population of 50 case subjects, giving ***n*_1_** = (50,50,50) and ***c*** = 0.70***n*_1_** = (35,35,35). Figure 2 gives the probability mass functions of 100,000 realisations of the three estimators 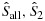 and 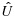. Note the slight negative skew evident in the distribution of 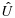. The empirical biases and MSEs were (0.0308, -0.0001, -0.0001) and (0.0024, 0.0042, 0.0033) respectively.

Table 1 shows the bias and MSE of the estimators for a range of further parameter values for ***S*** and ***n*_1_**, where *n*_2_=50 and ***c*** = 0.70***n*_1_** as before. *P*(continue) gives the probability that the whole trial continues to the validation stage, while *P*(best) is the probability that the biomarker with the highest (or joint-highest) sensitivity is selected for validation in stage 2, conditional on the trial actually continuing to the validation stage.

**Table 1:** Simulation results with *n*_2_=50 and *c* = 0.70*n*_1_. The mean bias and MSE shown are 100 times the actual estimates. There were 100,000 simulations for each set of parameter values.

				Bias (MSE)×100		
	Parameter values	*P*(continue)	*P*(best)	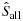	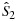	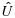
1.	***S*** = (0.50,0.70)	0.570	0.997	2.289	0.016	0.000
	***n*_1_** = (50,50)			(0.199)	(0.421)	(0.313)
2.	***S*** = (0.60,0.80)	0.914	0.906	1.097	−0.006	−0.003
	***n*_1_** = (15,25)			(0.222)	(0.336)	(0.267)
3.	***S*** = (0.50,0.70,0.70)	0.810	0.987	2.909	−0.005	−0.006
	***n*_1_** = (25,25,20)			(0.313)	(0.420)	(0.376)
4.	***S*** = (0.50,0.60,0.70,0.80)	0.985	0.807	1.400	−0.015	−0.001
	***n*_1_** = (30,40,40,40)			(0.197)	(0.340)	(0.244)
5.	***S*** = (0.58,0.60,0.62,0.64)	0.580	0.422	4.689	−0.005	0.010
	***n*_1_** = (40,35,30,30)			(0.426)	(0.466)	(0.418)
6.	***S*** = (0.70,0.70,0.70,0.70)	0.965	1	3.465	0.032	0.023
	***n*_1_** = (50,50,50,50)			(0.265)	(0.420)	(0.336)

The MLE 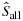 is biased high, and this bias is most pronounced for larger values of *K* and when the true sensitivities are similar. Note that 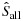 is still biased even when the probability of continuing to stage 2 is close to 100% (e.g. scenario 6). This indicates two sources of bias: the bias due to early stopping and the bias due to selecting the ‘best’ classifier from a set of candidates. The first source of bias would be expected to disappear when the probability of continuing to stage 2 is 100% but not the second.

The UMVCUE 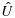 is unbiased as expected, and it also has a lower MSE than the unbiased estimator 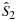 that only uses the stage 2 data. Indeed, there was a reduction of MSE ranging from 10% (for scenario 5) to 28% (for scenario 1). However, 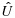 generally has a greater MSE than 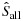, by up to 57% (for scenario 1). This is not always the case – for scenario 5, the large bias of 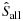 leads to a slightly greater MSE.

### 4.2 Interval estimation

We also consider the coverage of the confidence intervals constructed using the two procedures in Section 3.3, with *α* = 0.05. Table 2 shows the resulting mean coverage and confidence interval width for the scenarios in Table 1.

**Table 2:** Confidence interval simulation results with 10,000 simulated trials for each set of parameter values. As before, *n*_2_ = 50 and *c* = 0.70*n*_1_. The number of boot-strapped samples per simulation was set to *B*= 10,000. For comparison purposes, values for the optimised Sill–Sampson confidence intervals are shown in italics – note that only 1000 simulated trials were used here due to the computational cost.

					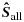				
		Sill–Sampson		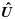 NP bootstrap		Clopper–Pearson		NP bootstrap	
	Parameter values	Coverage	CI width	Coverage	CI width	Coverage	CI width	Coverage	CI width
1.	***S*** = (0.50,0.70)	0.966	0.228	0.934	0.215	0.965	0.183	0.892	0.153
	***n*_1_** = (50,50)	*0.956*	*0.227*
2.	***S*** = (0.60,0.80)	0.969	0.214	0.945	0.196	0.966	0.193	0.938	0.169
	***n*_1_** = (15,25)	*0.950*	*0.211*
3.	***S*** = (0.70,0.70,0.70)	0.965	0.233	0.940	0.222	0.951	0.181	0.825	0.150
	***n*_1_** = (50,50,50)	*0.957*	*0.232*
4.	***S*** = (0.50,0.70,0.70)	0.968	0.249	0.941	0.236	0.949	0.219	0.885	0.188
	***n*_1_** = (25,25,20)	*0.962*	*0.248*
5.	***S*** = (0.50,0.60,0.70,0.80)	0.965	0.204	0.943	0.188	0.958	0.174	0.904	0.150
	***n*_1_** = (30,40,40,40)	*0.964*	*0.201*
6.	***S*** = (0.58,0.60,0.62,0.64)	0.961	0.262	0.943	0.256	0.913	0.212	0.731	0.180
	***n*_1_** = (40,35,30,30)	*0.958*	*0.262*
7.	***S*** = (0.70,0.70,0.70,0.70)	0.961	0.236	0.937	0.225	0.942	0.180	0.793	0.149
	***λ*** = (50,50,50,50)	*0.955*	*0.235*						

The coverage for the MLE 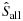 calculated using the nonparametric bootstrap is substantially lower than the nominal 95*%*, with values as low as 73% (for scenario 6). In contrast, the bootstrap coverage of the UMVCUE 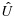 is much closer to the nominal, hovering around 94% for all the scenarios. The bootstrapped confidence interval widths are greater for 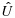 than for 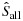, with an increase ranging from 16% (for scenario 2) up to 51% (for scenario 7).

Using exact (likelihood-based) approaches give better coverage for both the MLE and UMVCUE, at the cost of slightly wider confidence intervals. For the MLE, the Clopper–Pearson approach gives conservative coverage for the majority of the scenarios, except for the last two sets of parameter values where the coverage was less than the nominal 95*%*. In contrast, the Sill–Sampson approach gives conservative confidence intervals for all the parameter values considered. This results in an increase in confidence interval width ranging from 11% (for scenario 2) up to 31% (for scenario 7).

Using optimised Sill–Sampson confidence intervals gives a slight reduction in width and coverage, although the latter is still above 95% in all the scenarios. However, this comes at a much greater computational cost when simulating a large number of trials. Hence, we do not consider optimised Sill–Sampson confidence intervals any further in this research article.

### 4.3 Hypothesis testing

Consider now testing the hypothesis *H*_0_:*s*_*M*_≤s_*_ versus *H*_1_:*s*_*M*_>s_*_, using exact 95% one-sided confidence intervals. We compare using Clopper–Pearson confidence intervals for 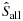 with the Sill–Sampson approach, where *H*_0_ is rejected if s_*_ is less than the lower bound of the confidence interval. For a given set of true sensitivities ***S***, let ***S*_0_**={*s*∈***S***:*s*≤s_*_}. Then we define the conditional type I error rate as *α* = *P*(reject *H*_0_|*s*_*M*_∈***S*_0_**,*Q*). The unconditional type I error rate is defined as *P*(reject *H*_0_,*s*_*M*_∈***S*_0_**), where there is no conditioning on continuing to stage 2.

Similarly, the conditional power of the test is defined as *P*(reject *H*_0_|*s*_*M*_∈***S***∖***S*_0_**,*Q*). The unconditional power is *P*(reject *H*_0_,*s*_*M*_∈***S***∖***S*_0_**), with no conditioning on continuing to stage 2.

**Figure 3:**
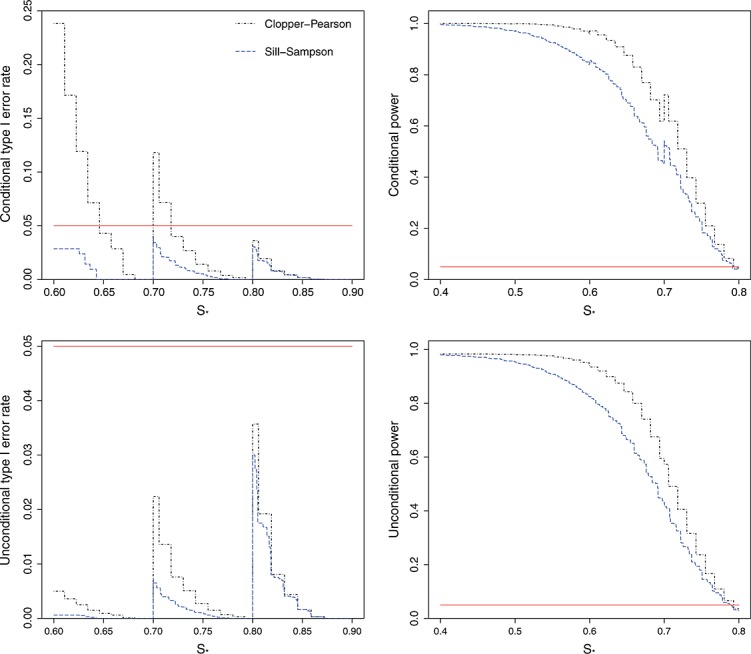
Conditional and unconditional type I error rates and power for testing the hypothesis *H*_0_:*s*_*M*_≤s_*_ versus *H*_1_:*s*_*M*_>s_*_, using exact 95% one-sided confidence intervals. The true sensitivities are constrained to the set *S* = (0.50,0.60,0.70,0.80), with stage 1 sample sizes *λ* = (30,40,40,40). Plots show the results from 10,000 simulated sets of trial data. The horizontal line shows the nominal 5% level.

Figure 3 shows the conditional and unconditional type I error rates and powers when the sensitivities are constrained to the set ***S*** = (0.50,0.60,0.70,0.80), with stage 1 sample sizes ***λ*** = (30,40,40,40). Using the Clopper–Pearson confidence intervals for 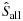 can give highly inflated conditional type I error rates (as high as 24%), particularly for values of s_*_ that are just above 0.60 or 0.70.

In contrast, using the Sill–Sampson approach guarantees that the conditional type I error rate will be less than 5% for all values of s_*_. This comes at the cost of lower power, both conditionally and unconditionally. Note that while using exact confidence intervals for the MLE does not control the type I error conditionally, it does control it unconditionally since *P*(*s*_*M*_∈***S*_0_**) is low when s_*_<0.70.

**Figure 4:**
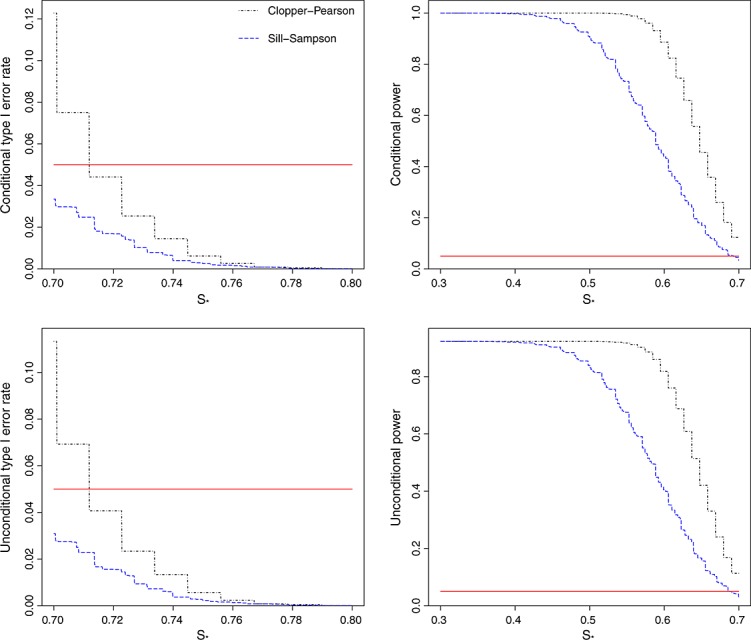
Conditional and unconditional type I error rates and power for testing the hypothesis *H*_0_:*s*_*M*_≤s_*_ versus *H*_1_:*s*_*M*_>s_*_, using exact 95% one-sided confidence intervals. The true sensitivities are constrained to the set *S* = (0.70,0.70,0.70), with stage 1 sample sizes *λ* = (50,50,50). Plots show the results from 10,000 simulated sets of trial data. The horizontal line shows the nominal 5% level.

Figure 4 shows the conditional and unconditional type I error rates and powers for the scenario ***S*** = (0.70,0.70,0.70) and ***λ*** = (50,50,50). This time, using the confidence intervals for 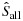 gives inflated type I error rates both conditionally and unconditionally. Even unconditionally, the type I error rate can be as high as 11%. In contrast, the Sill–Sampson approach again guarantees that the type I error rate will be less than the nominal 5%. However, this is at the cost of a substantial loss of power compared with using the MLE.

## 5. Application to the family history questionnaire study

In this section, we return to the motivating example of the two-stage FHQ study by Walter *et al*. 19. Although a *χ*^2^-test for concordance was carried out before pooling data from the two stages, a natural question to ask is whether any bias was induced into the results by the stage 1 selection rules. Using the framework for bias adjusted inference outlined in Section 3, we calculate the UMVCUE and exact confidence intervals for the sensitivities of the selected questions.

### 5.1 Model description for the family history questionnaire

We use a slightly simplified version of the study design formulated in Section 3.1. Note that this model does not consider combinations of questions; hence, steps 3 and 4 in stage 1 are ignored. In the discussion, we comment on how the approach could potentially be extended to consider combinations of questions. In what follows, the focus is on estimating the sensitivity of the selected questions. The model for estimating the specificity, or other measures of diagnostic performance, will be very similar.

In the first stage, *K* questions are assessed on a case-control population, with the results for the *i*^th^ question available on *n*_1*i*_ cases and *m*_1*i*_ controls. Let *X*_*i*_ denote the number of true positives (TP) for the *i*^th^ question (*i* = 1,…,*K*). That is, the total number of ‘yes’ responses from the case population. Then the *X*_*i*_ are assumed to follow independent binomial populations: *X*_*i*_∼Bin(*n*_1*i*_,*s*_*i*_), where *s*_*i*_ denotes the true sensitivity of question *i*. In Section 5.3.2 we explore the performance of the method when this independence assumption in violated, as was the case (to a very limited extent) with the FHQ data.

It is worth noting that when analysing the sensitivity of the selected questions, we are explicitly conditioning on the specificity results (i.e. the number of false positives and true negatives) in addition to what was specified in Section 3.2. We use this fact for both of the selection procedures: Fisher's exact test and ranking by balanced accuracy.

#### 5.1.1 Fisher's exact test cut-off

Firstly, Fisher's exact test is applied to the contingency table given in Table 3 where FP_*i*_ = number of false positives and TN_*i*_ = number of true negatives for question *i*. As the focus is in estimating the sensitivity and we are conditioning on the observed specificity results from the trial, then the values of FP_*i*_ and TN_*i*_ are considered fixed for each *i*.

**Table 3:** Contingency table for Fisher's exact test.

		Question *i*	
		‘Yes’ = 1	‘No’ = 0
Increased risk	1	*X*_*i*_	*n*_1*i*_−*X*_*i*_
No increased risk	0	FP_*i*_	TNs_*i*_

The aim is to find the threshold that *X*_*i*_ must pass in order for Fisher's exact test to give a *p*-value *p*_*i*_<0.05. That is, the value of *c*_*i*_ such that 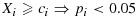. Because the FP_*i*_ and TN_*i*_ are fixed, then we can do so by simply setting *c*_*i*_ as the smallest value in {0,1,…,*n*_1*i*_} such that *p*_*i*_<0.05 for all 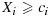.

Note that the conditioning on the observed number of false positives and true negatives is important. Indeed, another way of finding the Fisher's exact test threshold for the *X*_*i*_ would be to only consider the row and column totals as fixed, hence, allowing FP_*i*_ and TN_*i*_ to vary also. However, this would induce dependence between the *X*_*i*_ and the *c*_*i*_, which would invalidate the derived form of the UMVCUE.

Although a two-sided Fisher's exact test was used in a study, we did not have to consider departures towards the other extreme – i.e. values of *X*_*i*_≪*b*_*i*_ that gave *p*_*i*_<0.05. This was because all of the significant questions in the study actually passed the upper threshold *c*_*i*_. In addition, we would not be interested in a question that had especially low values of *X*_*i*_, because this would imply a low sensitivity. The balanced accuracy ranking (see the succeeding paragraphs) should rule out such questions being carried forward to stage 2.

In summary, for each *i*∈{1,…,*K*}, there is an associated fixed threshold *c*_*i*_. If 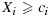 then Fisher's exact test will give a *p*-value <0.05; thus, *X*_*i*_ will be considered further in the balanced accuracy ranking.

Suppose *L* > 0 questions are identified as significant. Let 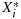 (*i* = 1,…,*L*) denote the number of true positives, where the relabelling preserves the order of the original labelling.

#### 5.1.2 Balanced accuracy ranking

The significant question with the greatest balanced accuracy is now selected. If there is a tie (which did not occur in the study data), we assume that the question with the smallest index would be chosen.

Now, suppose question *i* has a greater balanced accuracy than question *j*. This implies the following inequality on 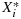 and 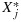:

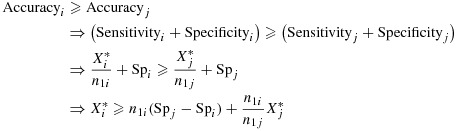
17 where 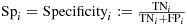.

Let (*i*_1_,*i*_2_,…,*i*_*L*_) denote the vector of indices of the 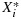 after they have been ordered by balanced accuracy, and let *M* = *i*_1_. Then from equation  the following inequality holds:

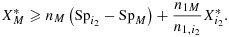
18

In the second stage, we test the selected question *M* from stage 1 on *n*_2_ additional cases and *m*_2_ additional controls. Let *Y* denote the number of true positives recorded in stage 2. Note that *Y* ∼ Bin(*n*_2_,*s*_*M*_), and is independent of *X*_*M*_.

### 5.2 The uniformly minimum variance conditionally unbiased estimator

To find the UMVCUE for the sensitivity *s*_*M*_ of the selected question (after the end of stage 2), we use equation , where

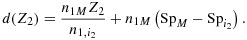
 Equation  holds when the number of significant questions *L* satisfies *L* > 1, which is what occurred in this study for all of the diseases considered.

### 5.3 Results

We now apply our results to the trial data from the FHQ study, first repeating the analysis carried out in the work of Walter *et al*., 19. Fisher's exact test indicated that an increased risk of diabetes was associated with questions 1 and 3 (*p* = 0.004 and *p* < 0.001). For IHD, questions 1, 2, 3 and 8 were significant (*p* = 0.013,*p* < 0.001,*p* = 0.018 and *p* = 0.048). For breast cancer (females only), there was a significant association for questions 6, 7, 8, 12a and 12b (*p* < 0.001,*p* < 0.001,*p* < 0.001,*p* < 0.001 and *p* = 0.002). Finally, increased risk of colorectal cancer was associated with questions 10 and 11 (*p* < 0.001 for both).

Table 4 shows the sensitivities, Fisher's exact test thresholds (FT) and balanced accuracies for each question. The questions that passed the Fisher threshold are shown in bold, with the ultimately selected question also boxed. If the significant question with the highest balanced accuracy is chosen, then question 3 is selected for diabetes, question 2 for IHD, question 8 for breast cancer and question 10 for colorectal cancer.

**Table 4:** Stage 1 sensitivities, Fisher's exact test thresholds (FT) and balanced accuracies for each condition. Questions passing the FT are shown in bold, with the selected question also boxed.

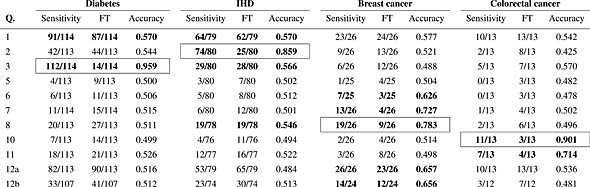

#### 5.3.1 Uniformly minimum variance conditionally unbiased estimator for the selected questions

Using the data from stages 1 and 2, we now calculate the value of the UMVCUE 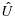 for the sensitivity of the selected question for each condition, and compare it with the various naïve estimators of the sensitivity 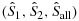. 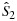 and 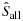 are defined as before, while 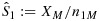 is the estimated sensitivity just using the stage 1 data.

Table 5 gives the values of the estimators for each disease, along with exact (likelihood-based) two-sided 95% confidence intervals. For 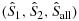, Clopper–Pearson confidence intervals are used, while the Sill–Sampson confidence interval is shown for 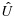.

**Table 5:** Uniformly minimum variance conditionally unbiased estimators (UMVCUE) and naïve estimators for the selected questions for each disease, with exact (likelihood-based) 95% confidence intervals.

Condition	Question	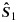	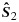	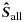	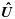
Diabetes	3	0.982	0.970	0.977	0.977
		(0.938, 0.998)	(0.914, 0.994)	(0.946, 0.992)	(0.946, 0.992)
Ischaemic heart disease	2	0.925	0.931	0.928	0.928
		(0.844, 0.972)	(0.845, 0.977)	(0.874, 0.963)	(0.874, 0.963)
Breast	8	0.731	0.636	0.688	0.662
cancer		(0.522, 0.884)	(0.407, 0.828)	(0.537, 0.813)	(0.455, 0.806)
Colorectal	10	0.846	0.750	0.800	0.800
cancer		(0.546, 0.981)	(0.428, 0.945)	(0.593, 0.932)	(0.579, 0.932)

For diabetes, IHD and colorectal cancer, the UMVCUE is *identical* to the MLE 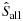 that uses data from both stages. This is a consequence of the formula for 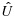 as described earlier. In addition, the Sill–Sampson confidence intervals for diabetes and IHD are virtually identical to the Clopper–Pearson intervals for 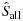. This is an attractive feature: the approach is able to identify when selection bias is *not* an issue.

**Figure 5:**
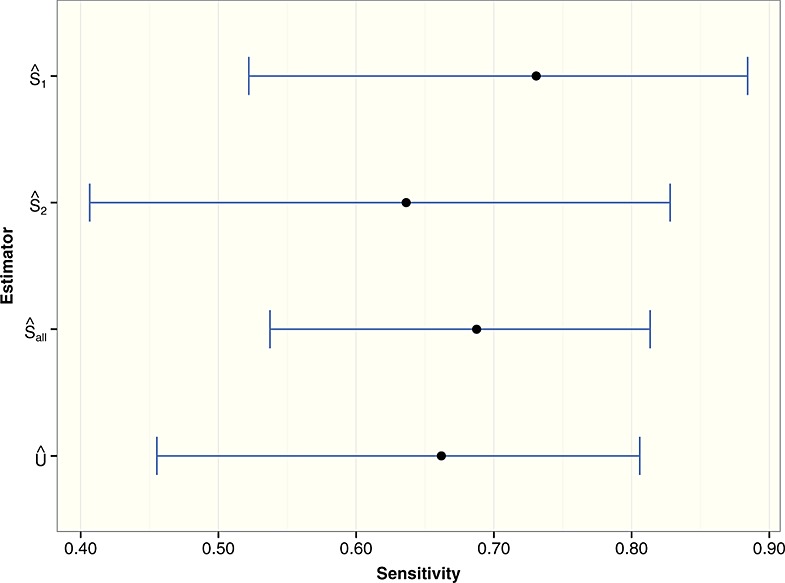
Plot of point estimates and exact (likelihood-based) 95% confidence intervals for the breast cancer data.

However, for breast cancer, the UMVCUE is smaller than 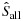. Looking at the individual estimates for the stages 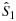 and 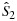, there is an especially large relative drop from 0.731 to 0.636 between stages 1 and 2, which supports the idea that the stage 1 data was biased high by the selection criteria. Figure 5 gives a graphical representation of the breast cancer data.

If we follow Walter *et al*. 19 and use Pearson's *χ*^2^-test to compare the sensitivity between the two stages, the *p*-values are 0.873, 1.000, 0.696 and 0.920 for diabetes, IHD, breast cancer and colorectal cancer, respectively. It is interesting to note that the *p*-value for breast cancer is substantially lower than those for the other diseases, although it is still far above 0.05. This suggests that the *χ*^2^-test is too conservative as a tool for detecting bias in the stage 1 data.

Indeed, for breast cancer, suppose we assume that the stage 1 data as well as the total number of cases in stage 2 are fixed. Then the number of true positives in stage 2 would have to be less than or equal to 8 (i.e. a sensitivity less than 0.363) in order for the *χ*^2^-test to reject the null hypothesis.

#### 5.3.2 Correlation

Finally, we consider the effect of correlation on the sensitivity estimates for the FHQ data. Recall that the data were assumed to be drawn from independent populations. However, in the FHQ study, each participant answered multiple questions. It sometimes happens that the answer to one questions should (logically at least) determine the answer to another question as well. For example, answering ‘yes’ to question 12b should mean that the answer to question 12a will also be ‘yes’. For these two reasons, we might expect there to be some correlation between the sensitivity estimates for different questions. The correlation matrix (using pairwise-complete responses) for all of the stage 1 data is displayed in Table 6.

**Table 6:** Correlation matrix for all of the stage 1 data in the family history questionnaire study, using pairwise-complete responses.

	Q1	Q2	Q3	Q5	Q6	Q7	Q8	Q10	Q11	Q12a	Q12b
Q1	1	0.142	0.121	0.051	0.072	0.028	0.096	0.047	0.086	0.049	0.062
Q2	0.142	1	0.101	0.090	0.056	0.033	0.062	0.006	0.050	0.002	0.063
Q3	0.121	0.101	1	−0.003	0.067	0.027	0.013	0.005	0.069	0.022	0.029
Q5	0.051	0.090	−0.003	1	0.006	0.016	0.014	−0.049	−0.014	0.121	0.012
Q6	0.072	0.056	0.067	0.006	1	0.005	0.018	0.045	−0.002	0.085	0.096
Q7	0.028	0.033	0.027	0.016	0.005	1	0.282	0.004	−0.007	0.116	0.124
Q8	0.096	0.062	0.013	0.014	0.018	0.282	1	−0.023	0.056	0.240	0.112
Q10	0.047	0.006	0.005	−0.049	0.045	0.004	−0.023	1	0.193	0.084	0.112
Q11	0.086	0.050	0.069	−0.014	−0.002	−0.007	0.056	0.193	1	0.187	0.121
Q12a	0.049	0.002	0.022	0.121	0.085	0.116	0.240	0.084	0.187	1	0.395
Q12b	0.062	0.063	0.029	0.012	0.096	0.124	0.112	0.112	0.121	0.395	1

Reassuringly, the correlations between all of the questions appears to be rather small, with a mean (absolute) pairwise correlation of just 0.07. The maximum correlation coefficient was 0.395, for the pair (Q12a, Q12b), which is explained by the aforementioned reason.

Nevertheless, we simulated FHQ-like data with the above correlation structure, using a modified version of the R package bindata 21. The true sensitivities were assumed equal to the estimated stage 1 sensitivities, with 50,000 simulated data sets for each condition. For breast cancer the UMVCUE had a mean bias of -0.0082, which is less than 32% of the observed correction to the MLE for the actual FHQ data. For the other diseases there was no appreciable bias.

## 6. Discussion

In this research article, we present a framework for conditional estimation for a general two-stage trial design with binary classifiers. By allowing for generalised selection rules and arbitrary futility thresholds, our estimation strategy can be applied to a wide range of two-stage validation study designs. In particular, complex ranking criteria can be reverse engineered to fit within our framework.

We showed that using the usual MLE can lead to substantial conditional bias, especially when there are many candidate classifiers under consideration with similar true sensitivities. In contrast, the UMVCUE is indeed unbiased but often at the expense of a larger MSE. However, there are still large savings in efficiency when compared with just using the unbiased stage 2 data.

The usual MLE also can suffer from incorrect confidence interval coverage and inflated type I error rates for hypothesis testing, both conditionally and unconditionally. These issues can be avoided by using the Sill–Sampson approach to find exact confidence intervals, although this comes at the cost of reduced power. Although this approach is somewhat conservative, when presenting the results of a trial to a regulatory authority, any inflation in the type I error rate above the advertised level is likely to be deemed unacceptable 16.

The application of our inferential technique to the FHQ data demonstrated how the UMVCUE can identify whether selection bias is an issue. Point estimates for the selected questions using the UMVCUE and the MLE were identical for three of the conditions, with virtually identical confidence intervals as well. However, for breast cancer, the UMVCUE was able to identify and correct for the bias induced in the MLE. We also found that with the correlation structure present in the FHQ data, these results were not significantly affected by the minor violations of the independence assumption.

Our focus in this research article was in deriving unbiased estimators for the true sensitivity of the chosen classifier. However, by relaxing the unbiasedness condition slightly, it may be possible to achieve a lower MSE. One approach we tried was to use *median unbiased estimates*, as described by Jovic and Whitehead 22. Briefly, using the distribution functions *p*_1_(*s*_*M*_) and *p*_2_(*s*_*M*_) defined for the Sill–Sampson approach, the (approximate) median unbiased estimator is given by 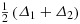, where *p*_2_(*Δ*_1_) = 0.5 and *p*_1_(*Δ*_2_) = 0.5. However, we found that there was no gain over the UMVCUE in terms of MSE, and the estimator was indeed biased slightly low in its mean.

We only considered a design that selects and evaluates the performance of a single classifier. However, many studies (including the FHQ study) *combine* multiple classifiers into *risk prediction models*. Much further research is needed to explore conditional estimation for combinations of classifiers, especially given the wide variety of model selection and validation procedures present in the literature. For example, recent work by Koopmeiners *et al*. 23 describes the issue of testing and validating a panel of biomarkers. Accounting for correlation will clearly be essential here too.

One way to try and deal with correlated classifiers is to decorrelate the variables of interest, as described by Zuber and Strimmer 24 in the context of biomarker discovery and gene-ranking by *t*-scores. However, is not clear whether similar transformations can be applied to binary data without altering its distribution.

A related issue would be to consider *joint* inference on the sensitivity and specificity. As mentioned in Section 5, by conditioning on the observed specificity results we treated the number of true negatives (and false positives) as fixed values. If we instead considered the number of true negatives as a binomial random variable (possibly correlated with the number of true positives), then further work would be needed to allow conditionally unbiased estimation. A complicating factor would be determining how the ranking criterion and ‘fixed’ thresholds change as the number of true positives and negatives are jointly varied.

Finally, another extension would be to consider inference trials with more than two stages. Bowden and Glimm 11 describe conditionally unbiased estimates for normally distributed outcomes with multiple stages of selection, and their approach could be extended to the binomial setting.

## Appendix

### Appendix A

#### A.1. Proof of the completeness and sufficiency of *Z*

Here we prove the following theorems (originally theorems 2.1 and 3.1 in the work of Tappin  18).

#### Theorem A.1

The statistic ***Z*** = (*Z*_1_,*Z*_2_,…,*Z*_2*L*_) is sufficient for (*s*_1_,*s*_2_,…,*s*_*L*_), where

#### Proof

The joint distribution of ***X***,*Y* is as follows:

Thus according to the factorisation criteria, the statistic ***Z*** is sufficient for (*s*_1_,*s*_2_,…,*s*_*L*_). □□

#### Theorem A.2

When ties are broken by selecting the population with the smallest index, the sufficient statistic ***Z*** is also complete.

#### Proof

Following Tappin 18 and Jung and Kim 25, we prove the result for *L* = 2 classifiers, and note that the argument easily extends to arbitrary *L* > 2.

For an arbitrary function *g*(·), defined on the range of ***Z***, we will show that *E*_***s***_[*g*(***z***)] ≡ 0 for all ***s***⇒*g*(***z***) ≡ 0. Without loss of generality, we assume that . Let

Note that *Z*_1_=*X*_*M*_,*Z*_2_=*X**i*_2_,*Z*_3_=*M* = *i*_1_ and *Z*_4_=*i*_2_. Because *Z*_1_=*X*_*M*_+*Y*, then the distribution of (***X***,*Z*_1_) is given by

We can now find the distribution of ***Z*** by summing over the support of *X*_*M*_:

where

and

Thus

27

Let  and *Q*(***s***,*j*,*l*):=*h*(***s***)/(1 − *s*_*j*_)^*l*^ for *j*∈{1,2}. Each term, say term *i*, in equation  has the factor  for some non-negative integers *k*_1*i*_,*k*_2*i*_,*l*_1*i*_,*l*_2*i*_. Because all terms have different factors, that is, (*k*_1*i*_,*k*_2*i*_,*l*_1*i*_,*l*_2*i*_) ≠ (*k*_1*j*_,*k*_2*j*_,*l*_1*j*_,*l*_2*j*_) for *i* ≠ *j*, any subset of the terms in equation  has a unique minimum among {*k*_1*i*_,*k*_2*i*_,*l*_1*i*_,*l*_2*i*_}.

On the one hand, if {*k*_1*i*_} has a unique minimum *k*_1_ and because *P*(***s***,1,*k*_1_) = 0 for all ***s***, letting *s*_1_→0 and *s*_2_>0 show that *g*(***z***) = 0, where *g*(***z***) is the coefficient of the term with the  factor. Similarly, if {*k*_2*i*_} has a unique minimum *k*_2_ and because *P*(***s***,2,*k*_2_) = 0 for all ***s***, letting *s*_2_→0 and *s*_1_>0 show that *g*(***z***) = 0, where *g*(***z***) is the coefficient of the term with the  factor.

On the other hand, if {*l*_1*i*_} has a unique minimum *l*_1_ and because *Q*(***s***,1,*l*_1_) = 0 for all ***s***, letting *s*_1_→1 and *s*_2_>0 show *g*(***z***) = 0, where *g*(***z***) is the coefficient of the term with the  factor. Similarly, if {*l*_2*i*_} has a unique minimum *l*_2_ and because *Q*(***s***,2,*l*_2_) = 0 for all ***s***, letting *s*_2_→1 and *s*_1_>0 show *g*(***z***) = 0, where *g*(***z***) is the coefficient of the term with the  factor.

Whichever coefficient is 0, we remove that term from *h*(***s***) before the next step. We continue this procedure until all terms in equation  are removed, concluding that *g*(***z***) ≡ 0 for all ***z*** in the support of ***Z***. □□

#### A.2. Derivation of the Sill–Sampson approach

Defining ***X*** = (*X*_1_,…,*X*_*L*_), consider the joint distribution of ***X***,*Y*|*Q*:

where *K*(***s***), with ***s*** = (*s*_1_,…,*s*_*L*_), is the probability of observing the event *Q*, *I*_*Q*_(***X***) is the indicator function for *Q*, and

Because *Z*_1_=*X*_*M*_+*Y*, the distribution of ***X***,*Z*_1_|*Q* is given by

We can now find the distribution of the complete sufficient statistic ***Z*** conditional on *Q* by summing over the support of *X*_*M*_:

where  is the indicator function for  on  and

Then the distribution of ***X***_−1_ is

where

The conditional distribution used to find the confidence intervals is *f*_*Q*_(*Z*_1_|***X***_−1_) = *f*_*Q*_(*Z*_1_,***X***_−1_)/*f*_*Q*_(***X***_−1_). Hence,

where

is the normalising constant.
